# Modulation of distinct gut microbiota signatures in acute and chronic DSS-colitis by Bawei Huangqin enema in mice

**DOI:** 10.1016/j.isci.2025.113568

**Published:** 2025-09-12

**Authors:** Juncong Hu, Liming Zhang, Zhibin Wang, Lei Shi, Yang Zhang, Tangyou Mao, Xiaowei Chen, Wenjing Pei

**Affiliations:** 1 Beijing University of Chinese Medicine, Beijing, China; 2 Dongfang Hospital, Beijing University of Chinese Medicine, Beijing, China; 3 Medical Department, the Second Affiliated Hospital of Anhui University of Chinese Medicine, Hefei, China

**Keywords:** Microbiome, Omics

## Abstract

Intestinal microbiota plays a key role in ulcerative colitis (UC), and its composition may vary between the disease’s acute (AUC) and chronic (CUC) stages. Using dextran sulfate sodium-induced mice models of AUC and CUC, this study characterized their distinct microbial dysbiosis patterns and evaluated the efficacy of a traditional Chinese medicine formula, Bawei Huangqin Enema (BHE). BHE treatment effectively alleviated colonic inflammation in both models. This was achieved by remodeling the gut microbiota in a stage-specific manner and by enhancing intestinal barrier integrity through the upregulation of tight junction proteins Occludin and MUC2. These findings highlight the distinct microbial features of AUC versus CUC and suggest that BHE’s therapeutic potential derives from its dual regulation of the microbiome and intestinal barrier, supporting a multi-target approach for managing UC.

## Highlights

• Gut microbiota profiles differ between acute and chronic colitis models
• Bawei Huangqin Enema (BHE) is therapeutic in acute and chronic colitis
• BHE modulates gut microbiota and repairs the intestinal barrier

## Graphical abstract

**Figure:**
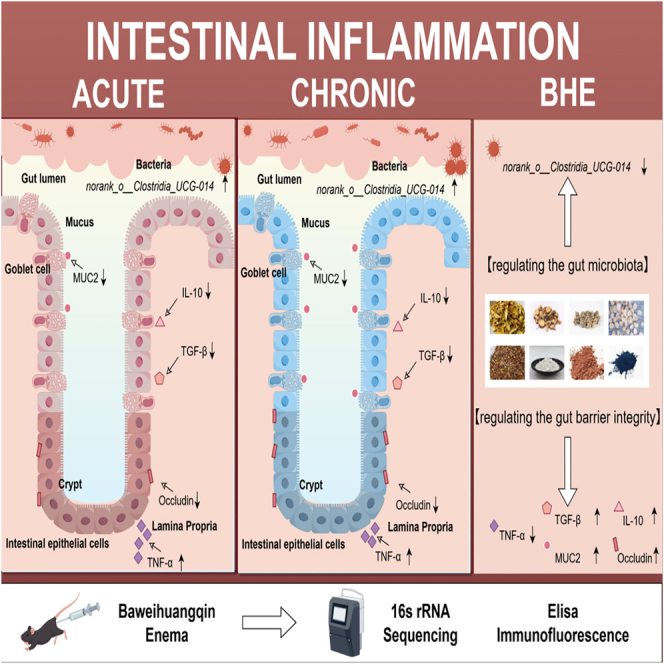

## Introduction

Ulcerative colitis (UC) is a long-term and recurring inflammatory bowel disease (IBD), and its incidence is on the rise globally.^1^ Epidemiological studies consistently indicate a rising incidence of UC in recent years, particularly in developed countries, with annual rates reaching up to 505 cases per 100,000 individuals in Europe.^2^ Current clinical management primarily involves pharmacological interventions, such as aminosalicylates, corticosteroids, and immunosuppressants, alongside surgical options for severe cases.^3^ However, these treatments are hindered by significant shortcomings; their effectiveness is often limited, they tend to produce a large number of adverse reactions, they fail to address the root cause of the condition, and there is still a considerable chance of recurrence of the disease.^4^

The gut microbiota, the vast collection of microorganisms in the gastrointestinal tract, is integral to maintaining intestinal homeostasis.^5^ A substantial body of research now emphasizes its crucial contribution to the pathogenesis of UC, where a state of dysbiosis—an imbalance in the microbial community—is a hallmark of the disease.^6^ This is often characterized by a depletion of beneficial microbes such as *Bifidobacterium*^7^*, Lactobacillus*,^8^ and a corresponding expansion of pro-inflammatory pathobionts, particularly within the *Proteobacteria.*^9^ Such microbial shifts can trigger aberrant immune responses, compromise the intestinal barrier, and perpetuate the inflammatory cascade central to UC.^10^

However, UC is not a monolithic disease; its clinical presentation is highly heterogeneous. It can manifest as acute ulcerative colitis (AUC), characterized by severe, debilitating inflammatory flares,^11^ or as chronic ulcerative colitis (CUC), defined by persistent low-grade inflammation, tissue remodeling, and a high risk of relapse.^12^ It is believed that differences in the composition and function of the gut microbiota between the two disease types are influenced by different levels of bacteria that promote or suppress inflammation.^13^ We hypothesize that these clinical and therapeutic disparities are underpinned by fundamentally different microbial states. In AUC, microbial communities undergo rapid changes, characterized by a sudden decrease in diversity and a significant proliferation of harmful bacteria such as *Shiga toxin-producing Escherichia coli*.^14^ Conversely, in CUC, the microbiota might adapt to the long-term inflamed environment, leading to the expansion of bacterial populations with heightened pathogenic potential that is inherently more challenging to reverse.^15^

Despite the clear clinical dichotomy between acute and chronic UC, there is a lack of studies that directly compare their corresponding gut microbiota profiles. A thorough characterization of these distinct microbial signatures is essential for understanding disease progression and developing more targeted, stage-specific therapies. To explore this, we investigated Bawei Huangqin Enema (BHE), a traditional Chinese medicine (TCM) formula containing *Scutellaria baicalensis charcoal (Chao huangqin), Sophora flavescens (Ku shen), Notoginseng powder (San qi), stir-fried Paeonia lactiflora (Chao baishao), Halloysitum rubrum (Chi shizhi), Flos Sophorae carbonisatus (Chao huaihua), Indigo Naturalis (Qing dai), and Margarita powder (Zhen zhufen)*. In TCM, herbal enemas are utilized to clear heat and dry dampness, a mechanism thought to improve drug absorption and efficacy within the inflamed intestine. This approach is further supported by multiple recent reports demonstrating that Chinese herbal medicine enema can regulate gut microbiota and intestinal inflammation, thereby inhibiting colitis.^16^^,^^17^ While BHE is presumed to have anti-inflammatory and immunoregulatory effects, the specific manner in which it modulates the gut microbiota in acute versus chronic UC models has remained unexplored. Therefore, this study aims to assess the therapeutic effect of BHE on mice models of acute and chronic UC, focusing on its impact on the distinct composition of the intestinal microbial community and gut barrier integrity in each state.

## Results

### BHE active ingredients and colitis status in acute ulcerative colitis and chronic ulcerative colitis mice

**Figure 1:**
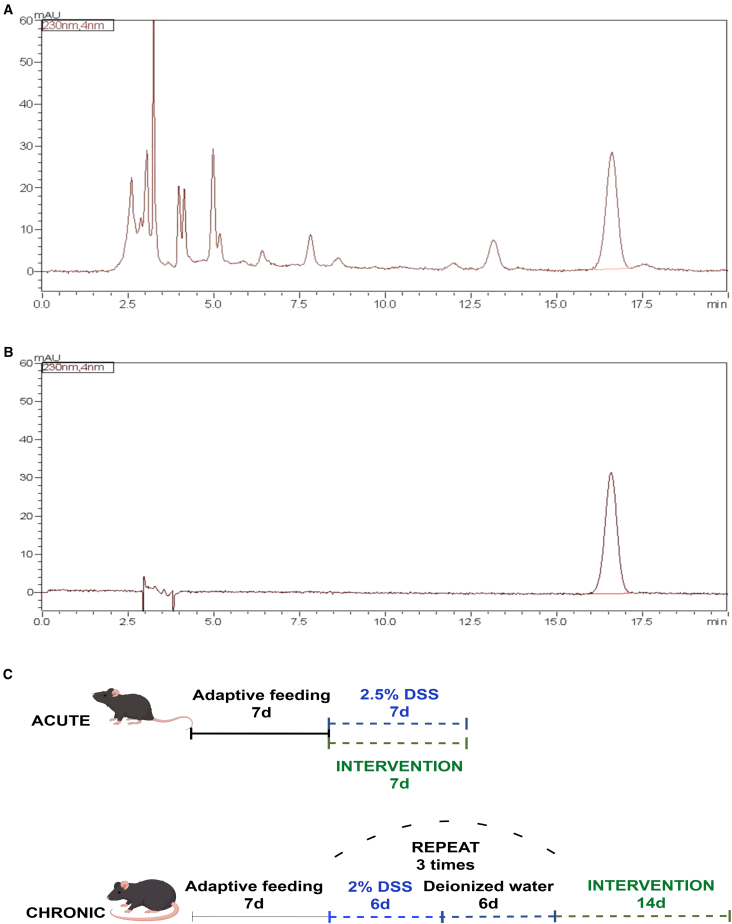
HPLC analysis of BHE and animal experiment design (A) HPLC analysis of BHE. (B) HPLC analysis of paeoniflorin. (C) Colitis was induced in acute (AUC) and chronic (CUC) mice models using distinct DSS protocols. The AUC model was established by administering 2.5% DSS in drinking water for one 7-day cycle, with the BHE intervention performed concurrently. The CUC model was induced by three cycles, each consisting of 6 days of 2% DSS followed by 6 days of deionized water. For the CUC model, the BHE intervention was administered after the completion of all DSS cycles. Detailed protocols are presented in the accompanying figure.

Initial high-performance liquid chromatography analysis determined that paeoniflorin is the main bioactive component present in white birch bark extract (BHE) (Figure 1A). Subsequently, AUC and CUC mice models of colitis were established (Figure 1B). No mice died during the modeling period, and histological examination (H&E staining) of colons from randomly sampled mice across all cages confirmed the presence of colonic inflammation.

**Figure 2:**
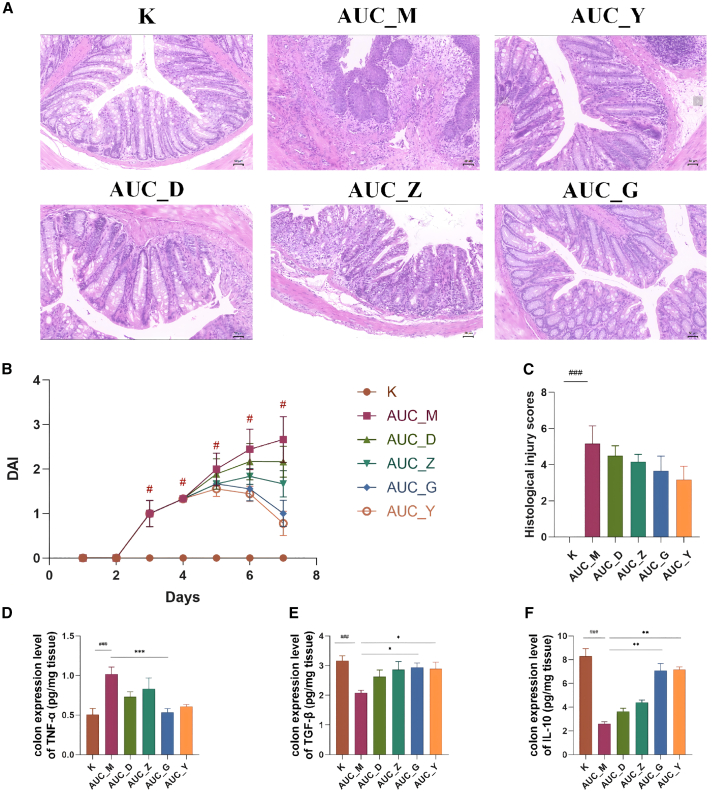
BHE ameliorates colitis in AUC mice (A) Typical colon tissue sections displayed with hematoxylin and eosin (H&E) staining (*n* = 3 per group; Scale bars: 50 μm). (B) The DAI scores of AUC mice (*n* = 6 per group). (C) The histological injury scores of AUC mice (*n* = 3 per group). (D–F) ELISA quantification of TNF-α (D), TGF-β (E), and IL-10 (F) levels in mice colon samples (*n* = 6 per group; values normalized to tissue weight). All data are presented as mean ± standard deviation (SD). Statistical significance was determined using the Kruskal-Wallis H test. Significance levels are indicated as follows: ^#^*p* < 0.05, ^###^*p* < 0.001 compared to the control group; ^∗^*p* < 0.05, ^∗∗^*p* < 0.01, ^∗∗∗^*p* < 0.001 compared to the AUC_M group. K, control group; AUC_M, acute colitis model group; AUC_Y, Mesalazine enema group; AUC_D, Low-Dose BHE group; AUC_Z, Median-Dose BHE group; AUC_G, High-Dose BHE group.

**Figure 3:**
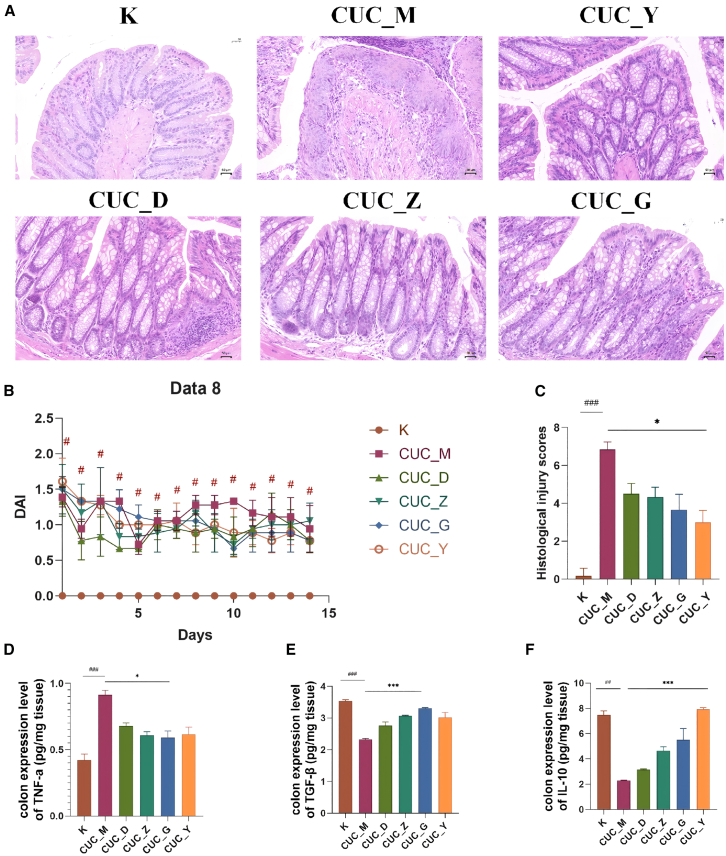
BHE ameliorates colitis in CUC mice (A) Typical colon tissue sections displayed with hematoxylin and eosin (H&E) staining (*n* = 3 per group; Scale bars: 50 μm). (B) The DAI scores of AUC mice (*n* = 6 per group). (C) The histological injury scores of AUC mice (*n* = 3 per group). (D–F) ELISA quantification of TNF-α (d), TGF-β (E), and IL-10 (F) levels in mice colon samples (*n* = 6 per group; values normalized to tissue weight). All data are presented as mean ± SD. Statistical comparisons were performed using the Kruskal-Wallis H test. Significance levels are indicated as follows: ^#^*p* < 0.05, ^###^*p* < 0.001 compared to the control group; ^∗^*p* < 0.05, ^∗∗∗^*p* < 0.001 compared to the CUC_M group. K, control group; CUC_M, chronic colitis model group; CUC_Y, Mesalazine enema group; CUC_D, Low-Dose BHE group; CUC_Z, Median-Dose BHE group; CUC_G, High-Dose BHE group.

Specifically, in AUC mice, severe colonic inflammation was observed. Macroscopically, this was characterized by significant colon shortening, marked wall thickening, and extensive, often confluent, ulcerations. Histologically, there was profound and widespread inflammatory cell infiltration extending deep into the submucosa, severe crypt destruction, significant goblet cell depletion, and extensive epithelial erosion and ulceration. In contrast, CUC mice exhibited a milder colitic phenotype. Macroscopically, colon shortening and wall thickening were less pronounced, and ulcerations were fewer or smaller and more discrete. Histologically, while inflammatory cell infiltration was present, it was generally less severe and more localized compared to AUC mice, with better preservation of crypt architecture and less extensive epithelial damage. Crucially, CUC mice also displayed focal or scattered areas of submucosal fibrosis, characterized by increased collagen deposition, which were not a prominent feature in the AUC group. This suggests a less acute but potentially more chronic or distinct reparative process in the CUC model (Figures 2A and 3A).

### Bawei Huangqin Enema modulates intestinal inflammation in acute ulcerative colitis and chronic ulcerative colitis mice

In this study, we established mice models of AUC and CUC and intervened with BHE at low, medium and high doses. To evaluate the severity of UC, we first conducted a Disease Activity Index (DAI) scoring assessment. As shown in Figure 2B, the results for the AUC model indicated that DAI scores did not change during the first two days of the intervention. However, scores increased significantly with prolonged DSS administration. The model group (AUC_M) reflected this change, exhibiting a significantly higher DAI score compared to the normal group (*p* < 0.05). This outcome is consistent with previously published findings.^18^ Histopathological examination of colon tissue from the AUC model revealed full-thickness mucosal ulceration and diffuse inflammatory infiltration, accompanied by a loss of goblet cells and crypt structures (Figure 2A). Furthermore, the analysis of histological injury scores showed a significant difference between the AUC_M and control groups (*p* < 0.001). Following BHE intervention, none of the BHE groups exhibited a significant decrease in the injury score compared to the AUC_M group (Figure 2C). In contrast, CUC mice exhibited milder colonic mucosal damage than AUC mice, characterized primarily by lymphocytic infiltration (Figure 3A). The overall DAI scores remained below 5, reflecting a state of low-grade chronic inflammation, with only the CUC_M group showing a significant difference compared to the control group (Figure 3B, *p* < 0.05). Furthermore, while the histological injury score of the CUC_M group was significantly higher than that of the control group (*p* < 0.001), only the CUC_Y group showed a significant difference in this score when compared to the CUC_M group *(p* < 0.05) (Figure 3C).

Specifically, within the AUC model, the K group (Control) exhibited healthy colonic tissue structure with intact and regularly arranged crypts, and no obvious inflammation or lesions, representing a normal or healthy state. The AUC_M group (Model) displayed severe tissue damage, extensive inflammatory infiltration, and structural disorganization, potentially accompanied by glandular hyperplasia or dysplasia, characterizing the disease model with significant pathological alterations. Compared to the AUC_M group, the AUC_D group showed some reduction in tissue damage, though inflammation and structural abnormalities remained evident. The AUC_Z group presented a similar level of improvement to AUC_D, yet did not fully recover to the healthy state of the K group. While the AUC_G group demonstrated improved tissue structure, significant inflammation and damage persisted. Notably, the AUC_Y group exhibited the most significant histological improvement, with colonic crypt structures largely restored to normal and reduced inflammation, closely approaching the healthy state of the K group. This restoration of colonic mucosal crypt structures was particularly evident in the high-dose BHE group (Figure 2A). Building upon BHE’s ability to ameliorate AUC colonic inflammatory pathology, we further investigated the expression levels of relevant inflammatory cytokines. Regarding colonic TNF-α expression, BHE significantly downregulated its levels, with the most pronounced reduction observed in the AUC_G group (*p* < 0.001), approaching the K group’s level (Figure 2D). Conversely, BHE upregulated colonic TGF-β expression, with significant increases noted in the AUC_G and AUC_Y groups (*p* < 0.05), also approaching the K group’s level (Figure 2E). Furthermore, BHE increased colonic IL-10 expression, with significant upregulation in both AUC_G and AUC_Y groups (*p* < 0.01) (Figure 2F).

Within the CUC model, the K group (Control) exhibited healthy colonic tissue structure with intact and regularly arranged crypts, and no obvious inflammation or lesions. In contrast, the CUC_M group (Model) displayed severe tissue damage, extensive inflammatory cell infiltration, and disorganized or even absent crypt structures, indicating significant chronic pathological alterations (Figure 3A). Compared with the CUC_M group, the CUC_Y group showed the most significant histological recovery, indicating that the colonic crypt structure had mostly returned to normal and that inflammation had been significantly reduced. The CUC_D group showed some reduction in tissue damage compared to CUC_M, though inflammation and structural abnormalities remained evident. The CUC_Z group presented a similar level of improvement to CUC_D, yet did not fully recover to the healthy state of the K group. While the CUC_G group demonstrated improved tissue structure, partial inflammation and damage persisted (Figure 3A). Building upon BHE’s ability to ameliorate CUC colonic inflammatory pathology, we further investigated the expression levels of relevant inflammatory cytokines (Figure 3A). Regarding colonic TNF-α expression, BHE significantly downregulated its levels, with the most pronounced reduction observed in the CUC_G group (*p* < 0.05), approaching the K group’s level (Figure 3D). Conversely, BHE upregulated colonic TGF-β expression, with significant increases noted in the CUC_G group (*p* < 0.001), also approaching the K group’s level (Figure 3E). Furthermore, BHE increased colonic IL-10 expression, with significant upregulation in the CUC_Y group (*p* < 0.001) (Figure 3F).

### Gut microbiota characteristics of acute ulcerative colitis and chronic ulcerative colitis mice models

**Figure 4:**
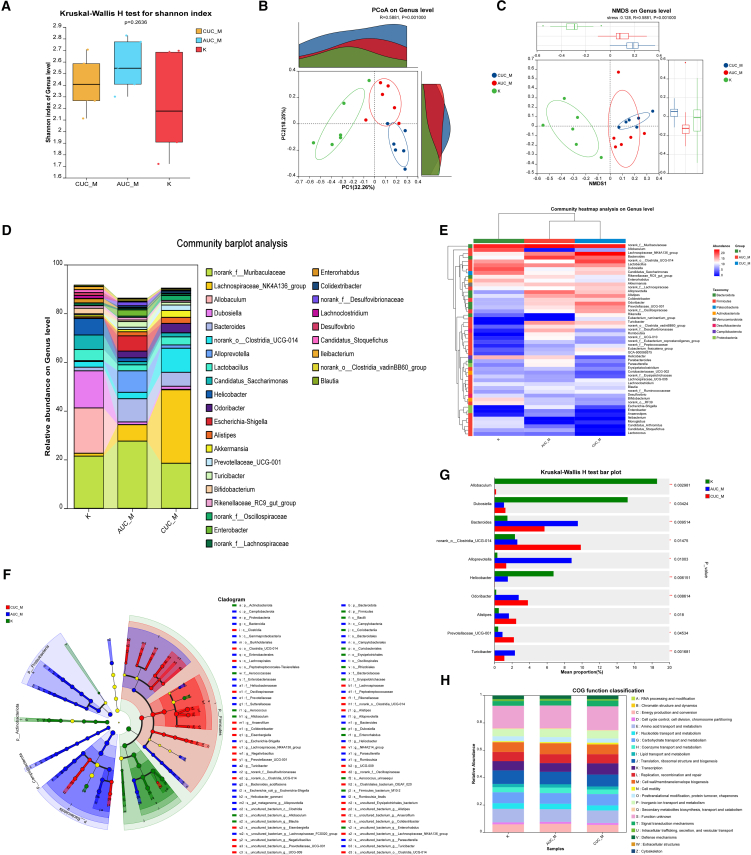
Analysis of the gut microbiota in control group, AUC and CUC mice models (*n* = 6 per group) (A) Comparative analysis of the Shannon index in the control group, AUC, and CUC mice models. (B) PCoA analysis on the genus level in the control group, AUC, and CUC mice models. (C) NMDS analysis on genus level in control group, AUC and CUC mice models. (D) The barplot of community analysis on the genus level in the control group, AUC, and CUC mice models. (E) The heatmap of community analysis on the genus level in the control group, AUC, and CUC mice models. (f) The LEfSe analysis on genus level in control group, AUC and CUC mice models. (G) The bar plot of Kruskal-Wallis H analysis on genus level in control group, AUC and CUC mice models. (H) The heatmap of predicted KO functions in the control group, AUC, and CUC mice models. K, control group; AUC_M, acute colitis model group; CUC_M, chronic colitis model group.

Following the observation of distinct colonic pathological damage in AUC and CUC mice models, we proceeded to investigate their gut microbiota characteristics. This comprehensive analysis mapped the gut microbial communities across three groups: K (healthy control), acute ulcerative colitis model (AUC_M), and chronic ulcerative colitis model (CUC_M). Although no significant differences were observed between populations in terms of alpha diversity, which measures species richness and homogeneity through the Shannon index (*p*-value 0.2636), as evidenced by the Chao index in Figure S1 (Figure 4A), beta diversity analysis (PCoA and NMDS) clearly demonstrated significant distinctions in microbial community structure among the three groups (Adonis R = 0.5881, *p* = 0.001; Anosim R = 0.5881, *p* = 0.001) (Figures 4B and 4C). Specifically, the microbial composition of the healthy control (K group) was markedly distinct from both disease model groups (AUC_M and CUC_M), and unique community characteristics were also observed between AUC_M and CUC_M (Figure 4D). At the genus level, the K group was primarily characterized by genera such as *Allobaculum, Dubosiella, and Helicobacter.* In the AUC_M group, the relative abundance of *norank_f_Muribaculaceae* and *Bacteroides* increased, while certain genera highly abundant in the K group (e.g., *Allobaculum and Dubosiella*) decreased. In the CUC_M group, the relative abundance of *norank_o__Clostridia_UCG-014* significantly increased, concurrent with a notable decrease in certain genera highly abundant in the K group (e.g., *Allobaculum and Dubosiella*) (Figure 4D).

The heatmap further illustrated that the K group was enriched in certain genera (e.g., *norank_f_Muribaculaceae*, *Allobaculum, and Dubosiella*). The AUC_M group showed the enrichment of genera distinct from both CUC_M and K groups (e.g., *Alloprevotella*). Meanwhile, the CUC_M group was enriched in yet another set of genera (e.g., *norank_o_Clostridia UCG-014, Lachnospiraceae NK4A136 group*) (Figure 4E). LEfSe analysis revealed that microorganisms enriched in the K group (indicated in green) included the families *f_Aerococcaceae*, along with genera such as *Aerococcus* and *Allobaculum*. These represent common dominant microbiota found in a healthy gut. The AUC_M group (indicated in blue) was enriched in microorganisms such as *Campilobacterota* and *Proteobacteria*. Conversely, the CUC_M group (indicated in red) showed the enrichment of microorganisms, including *Clostridia* and *Lachnospirales* (Figure 4F). Kruskal-Wallis H test results further indicated significant enrichment of *Bacteroids* and *Alloprevotella* in the AUC_M group (*p* < 0.01, *p* < 0.05). *Allobaculum* and *Dubosiella* were significantly enriched in the K group (*p* < 0.01, *p* < 0.05). *norank_o Clostridia UCG-014 and Prevotellaceae UCG-001* were significantly enriched in the CUC_M group (*p* < 0.01, *p* < 0.05) (Figure 4G). Finally, PICRUSt2 functional prediction analysis revealed specific functional enrichments: 'K: Transcription' was significantly enriched in the CUC group, while 'M: Cell wall/membrane/envelope biogenesis' showed significant enrichment in both AUC and CUC groups (Figure 4H).

### Bawei Huangqin Enema modulates the gut microbiota profiles in acute ulcerative colitis mice models

**Figure 5:**
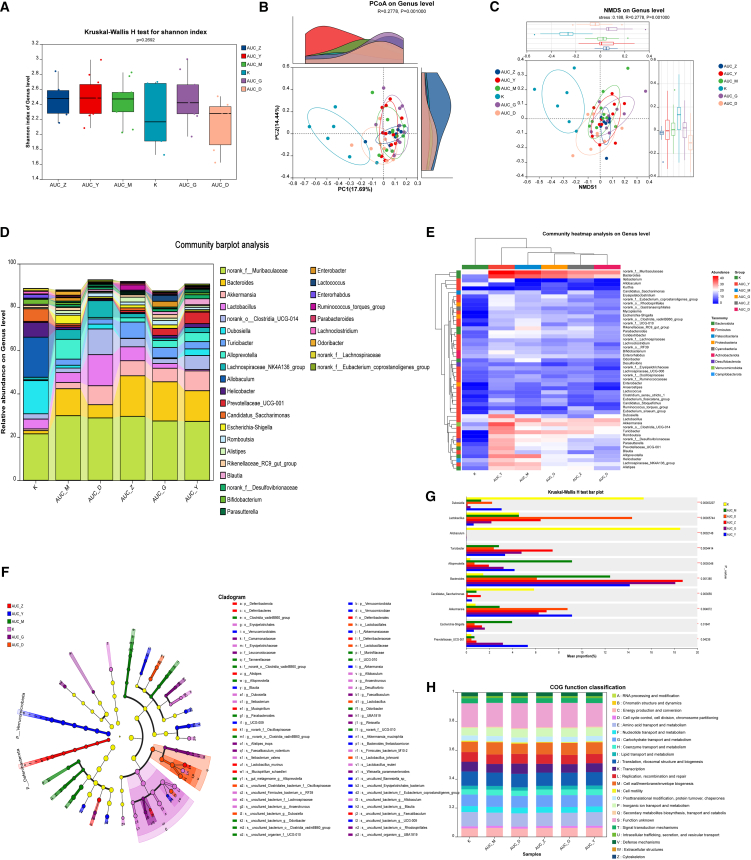
Effect of BHE on the gut microbiota in AUC mice models (*n* = 6 per group) (A) The Shannon diversity index was assessed for AUC mice models receiving different concentrations of BHE. (B) PCoA was performed on AUC mice receiving different doses of BHE at the genus classification level. (C) NMDS was performed at the genus level on AUC mice receiving different interventions. (D) Community composition histograms were used to show the differences in AUC mice at the genus level under different treatments. (E) Heat maps were used to depict the community structure of AUC mice at the genus level after different interventions. (F) LEfSe analysis was performed at the genus level to identify taxa with significant differences in abundance among different treatments of AUC mice. (G) Kruskal-Wallis H test histograms were used to show community differences at the genus level among different treatment groups. (H) Heat maps were used to demonstrate predicted KO functions of KEGG in AUC mice treated with different interventions. K, control group, AUC_M, acute colitis model group, AUC_Y, mesalazine enema group, AUC_D, low-dose BHE group, AUC_Z, medium-dose BHE group, AUC_G, high-dose BHE group.

Under conditions of BHE intervention for AUC-induced intestinal inflammation, we also investigated its impact on the gut microbiota composition in AUC mice. The results showed that there were no significant differences in alpha diversity between different groups as measured by the Shannon index. The Kruskar-Wallis H-test yielded a *p*-value of 0.2692, which is significantly higher than the traditional significance threshold of 0.05 (Figure 5A). This suggests that despite minor variations in the median and distribution range of Shannon indices, the within-sample species richness and evenness of the microbial communities were statistically similar across all groups. The Chao index is presented in Figure S2. However, beta diversity analysis (PCoA and NMDS) clearly demonstrated significant distinctions in microbial community structure among the three groups (Adonis R = 0.2778, *p* = 0.001; Anosim R = 0.2778, *p* = 0.001) (Figures 5B and 5C). At the genus level, the community bar chart (Figure 5D) showed that the K group was predominantly composed of beneficial bacteria such as *Dubosiella, Helicobacter* and *Candidatus_Saccharimonas.* In contrast, the AUC_M group exhibited a significant shift in composition, with a decrease in the abundance of some K-group dominant bacteria (e.g., *Allobaculum* and *Helicobacter*) and an increase in the relative abundance of genera such as *Escherichia-Shigella* and *norank_f__Muribaculaceae*. Notably, the microbial composition of the BHE intervention groups (AUC_D, AUC_Z, AUC_G) partially ameliorated the dysbiosis observed in the AUC_M group. The genus-level community heatmap (Figure 5E) further highlighted distinct high-abundance bacterial groups: the AUC_M group showed the unique enrichment of *norank_f_Muribaculaceae* and *Bacteroides*.

LEfSe analysis (Figure 5F) revealed specific microbial enrichments across groups: the K group (pink) was enriched in several health-associated taxa, including *Dubosiella, Ileibacterium, Allobaculum,* and so on. The AUC_M group (green) showed the enrichment of potentially disease-associated genera such as *Alloprevotella, Parabacteroides,* and *norank_f__UCG-010.* The AUC_Y group (blue) was enriched in *Blautia* and *Akkermansia.* The AUC_Z group (red) exhibited the enrichment of *Alistipes* and *Lactobacillus*. Furthermore, AUC_G (purple) and AUC_D (light red) displayed their own unique enriched bacterial groups, with AUC_G enriched in *Leuconostocaceae* and AUC_D in *Alistipes*. Further analysis using the Kruskal-Wallis H test (Figure 5G) identified specific genera with significant differential abundances among groups: *Alloprevotella* and *Escherichia-Shigella* in the AUC_M group (*p* < 0.001 and *p* < 0.05). Conversely, *Dubosiella* and *Allobaculum* were significantly enriched in the K group (*p* < 0.001). *Lactobacillus* were significantly increased in the AUC_D group (*p* < 0.001). *Turicibacter* showed significant elevation in the AUC_Z group (*p* < 0.001). *Prevotellaceae_UCG-001* was significantly enriched in the AUC_G and AUC_Y group (*p* < 0.05). Finally, PICRUSt2 functional prediction analysis indicated alterations in predicted functions, with “M: Cell wall/membrane/envelope biogenesis” and “K: Transcription” showing changes following BHE intervention (Figure 5H).

### Bawei Huangqin Enema modulates the gut microbiota profiles in chronic ulcerative colitis mice models

**Figure 6:**
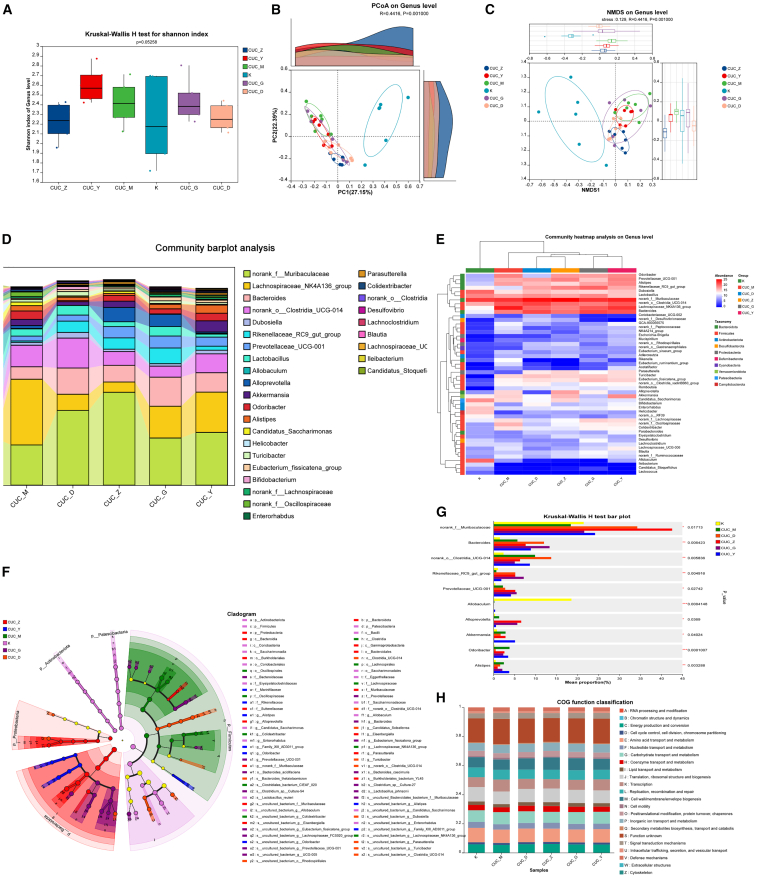
Effect of BHE on the gut microbiota in CUC mice models (*n* = 6 per group) (A) The Shannon diversity index was assessed for CUC mice models receiving different concentrations of BHE. (B) PCoA was performed on CUC mice receiving different doses of BHE at the genus classification level. (C) NMDS was performed at the genus level on CUC mice receiving different interventions. (D) Community composition histograms were used to show the differences in CUC mice at the genus level under different treatments. (E) Heat maps were used to depict the community structure of CUC mice at the genus level after different interventions. (F) LEfSe analysis was performed at the genus level to identify taxa with significant differences in abundance among different treatments of CUC mice. (G) Kruskal-Wallis H test histograms were used to show community differences at the genus level among different treatment groups. (H) Heat maps were used to demonstrate predicted KO functions of KEGG in CUC mice treated with different interventions. K, control group, CUC_M, chronic colitis model group, CUC_Y, mesalazine enema group, CUC_D, low-dose BHE group, CUC_Z, medium-dose BHE group, CUC_G, high-dose BHE group.

We also investigated the impact of BHE intervention on gut microbiota alterations in CUC mice. The Kruskal-Wallis H test for alpha diversity (Shannon index) yielded a *p*-value of 0.05258 (Figure 6A), indicating no significant differences in microbial community alpha diversity (i.e., within-sample species richness and evenness) among the various groups. The Chao index is available in Figure S3. However, beta diversity analysis (PCoA and NMDS) clearly demonstrated significant distinctions in microbial community structure among the three groups (Adonis R = 0.4416, *p* = 0.001; Anosim R = 0.4416, *p* = 0.001) (Figures 6B and 6C). At the genus level, the community bar chart (Figure 6D) showed that the K group was predominantly composed of bacteria such as *Allobaculum* and *Candidatus_Saccharimonas* which are typically considered important commensal bacteria in a healthy gut. In contrast, the CUC_M group exhibited a significant shift in composition: some K-group dominant bacteria (e.g., *Allobaculum*) decreased in abundance, while the relative abundance of genera such as *norank_o__Clostridia_UCG-014* appeared to increase. This clearly indicates that the disease model induced significant gut dysbiosis. The microbial composition of the BHE intervention groups (CUC_D, CUC_Z, CUC_G) displayed distinct characteristics, partially ameliorating the dysbiotic state observed in the CUC_M group. For instance, the proportions of *Rikenellaceae_RC9_gut_group* and *Lachnoclostridium* showed some recovery in the CUC_G and CUC_Y groups, and the CUC_G group exhibited a higher proportion of *Rikenellaceae_RC9_gut_group*. The genus-level community heatmap (Figure 6E) further highlighted distinct high-abundance bacterial groups: the K group was characterized by *Dubosiella, Candidatus_Saccharimonas,* and *Allobaculum.* The CUC_M group displayed the unique enrichment of *norank_f__Oscillospiraceae*. The intervention groups, in turn, exhibited patterns intermediate between the K and CUC_M groups, or their own distinct microbial enrichment patterns; for example, the abundance of *Rikenellaceae_RC9_gut_group* and *Eubacterium_fissicatena_group* in the CUC_G group was notably higher than in other groups.

LEfSe analysis (Figure 6F) further delineated specific microbial enrichments: the K group (purple) was enriched in several health-associated taxa, including *Candidatus_Saccharimonas* and *Enterorhabdus.* The CUC_M group (green) showed the enrichment of potentially disease-associated genera such as *Colidextribacter.* The CUC_Y group (blue) was enriched in *Alistipes, Family_XIII_AD3011_group, Odoribacter.* The CUC_Z group (red) exhibited the enrichment of *Alloprevotella*. CUC_G (purple) was enriched in *Candidatus_Saccharimonas*, and CUC_D (little red) in *norank_f_Muribaculaceae*. Furthermore, analysis of specific genera with significant differential abundances (Figure 6G) revealed that *norank_o__Clostridia_UCG-014* (*p* < 0.01) and *Odoribacter* (*p* < 0.001) were significantly elevated in CUC_M group. Conversely, *Allobaculum* (*p* < 0.001) was significantly enriched in the K group. *Rikenellaceae_RC9_gut_group* (*p* < 0.01) showed significant elevation in CUC_G group. *norank_o__Clostridia_UCG-014* and *Akkermansia* (*p* < 0.01*, p* < 0.05) were significantly increased in CUC_Y group. *norank_o__Clostridia_UCG-014* (*p* < 0.001) was significantly elevated in CUC_Z group, and *norank_f__Muribaculaceae* (*p* < 0.05) in CUC_D group. Finally, PICRUSt2 functional prediction analysis indicated that the predicted function “N: Cell motility” was altered following BHE intervention (Figure 6H).

### The effect of Bawei Huangqin Enema on the intestinal barrier in acute ulcerative colitis and chronic ulcerative colitis mice

**Figure 7:**
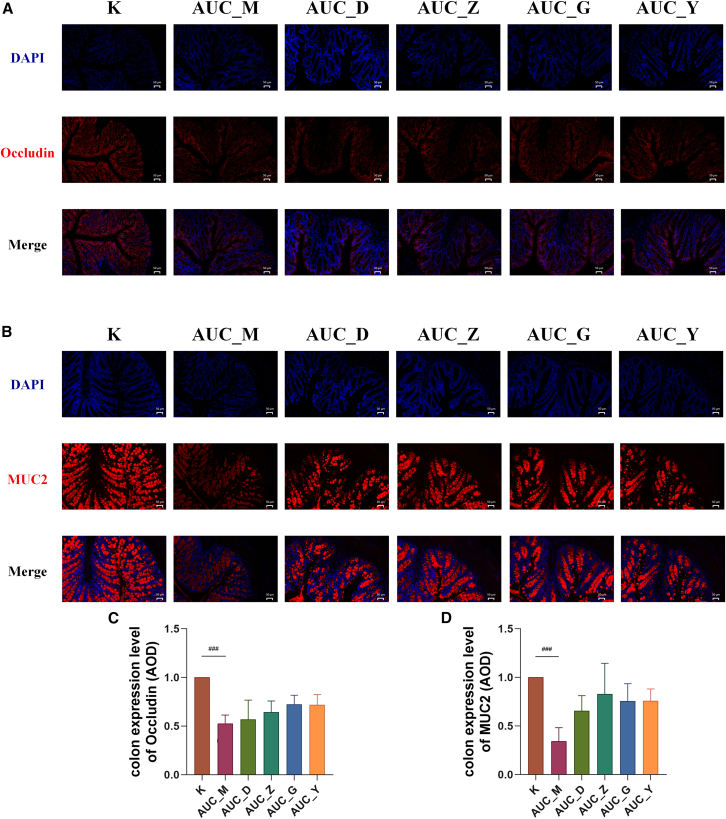
Effect of BHE on the intestinal barrier in AUC mice (A and B) Representative immunofluorescence images of mice colonic tissues. Sections were co-stained with DAPI (blue) and either (A) Occludin (red) or (B) MUC2 (red), and subsequently imaged using a fluorescence microscope (*n* = 6 per group; Scale bars: 50 μm). (C and D) Quantitative analysis of the average expression density of (C) colonic Occludin and (D) colonic MUC2 (*n* = 6 per group). All data are presented as mean ± SD. Statistical comparisons were performed using the Kruskal-Wallis H test. K, control group; AUC_M, acute colitis model group; AUC_Y, Mesalazine enema group; AUC_D, Low-Dose BHE group; AUC_Z, Median-Dose BHE group; AUC_G, High-Dose BHE group. Compared with the control group, ^###^*p* < 0.001.

**Figure 8:**
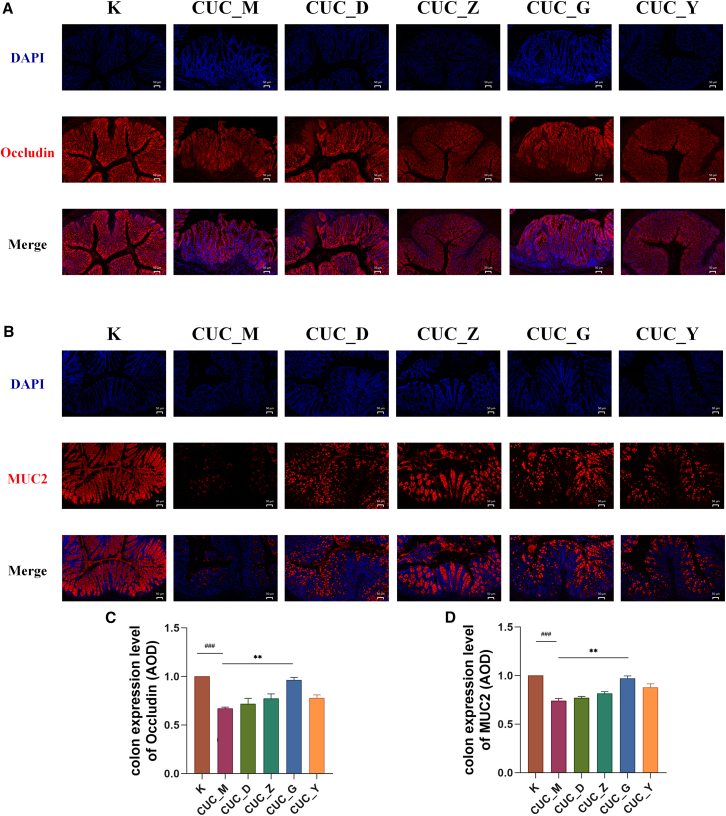
Effect of BHE on the intestinal barrier in CUC mice (A and B) Representative immunofluorescence images of mice colonic tissues. Sections were immunostained for (A) Occludin (red) or (B) MUC2 (red), and counterstained with DAPI (blue). Images were acquired using a fluorescence microscope (*n* = 6 per group; Scale bars: 50 μm). (C and D) Quantitative analysis of the average colon expression density for (C) Occludin and (D) MUC2 (*n* = 6 per group). All data are presented as mean ± SD. Statistical comparisons were performed using the Kruskal-Wallis H test. K, control group; CUC_M, chronic colitis model group; CUC_Y, Mesalazine enema group; CUC_D, low-dose BHE group; CUC_Z, median-dose BHE group; CUC_G, High-Dose BHE group. Compared with the control group, ^###^*p* < 0.001. Compared with CUC_M group, ^∗∗^*p* < 0.01.

Following BHE’s established role in modulating gut microbiota, we further examined its impact on the expression of intestinal mechanical barrier protein Occludin and mucin barrier protein MUC2 in AUC and CUC mice models. We observed a significant downregulation of Occludin and MUC2 expression in both AUC and CUC models (Figures 7 and 8). Immunofluorescence staining in the AUC model showed that BHE intervention increased Occludin (Figures 7A and 7C) and MUC2 (Figures 7B and 7D) expression, although this effect was not statistically significant. The AUC_G group’s expression levels were similar to those in AUC_Y group (Figure 7D). In contrast, BHE intervention in CUC models led to a statistically significant increase in Occludin (Figures 8A and 8C) and MUC2 (Figures 8B and 8D) expression, most notably in the CUC_G group.

## Discussion

UC is a prevalent chronic IBD globally, with relatively limited current treatment options.^19^ In recent years, there has been increasing interest in understanding how the gut microbiota affects this disease^20^; however, there is still a lack of in-depth comparative studies on the microbiota dynamics between AUC and CUC.^21^ Traditional treatment methods primarily focus on symptomatic relief, but their long-term curative effects are often unsatisfactory.^22^ In the field of UC research, the understanding of the gut microbiota-disease relationship is constantly evolving, and 16s rRNA sequencing technology provides an effective method for detecting microbial community distribution.^23^ Although previous studies have recognized the importance of gut microbiota in UC, most have focused solely on either acute or chronic UC, lacking comprehensive comparative analyses.

In this study, we successfully established murine models of acute (AUC) and chronic (CUC) colitis with distinct phenotypic differences (Figure 1B), which is significant for accurately simulating the varied clinical presentations of UC. We then evaluated the therapeutic effects of BHE, a TCM enema formulation in which paeoniflorin was identified as a key active constituent (Figure 1A). Overall, BHE visually appeared to alleviate colonic inflammation and promote mucosal damage recovery in both AUC and CUC mice (Figures 2A and 3A). However, a more detailed analysis of quantitative scores revealed a complex and paradoxical efficacy. Histological improvements were sparse; a significant benefit was observed only with the low dose in the acute model (AUC_D) (Figure 2C), whereas in the chronic model, only the Mesalazine control (CUC_Y) led to significant histological improvement (Figure 3C). Adding to this complexity, the high-dose chronic group (CUC_G) displayed a more favorable anti-inflammatory profile at the molecular level, showing superior regulation of tissue TNF-α, TGF-β, and IL-10 (Figures 3D–3F). This modulation of key cytokines is likely attributable to the anti-inflammatory properties of BHE components such as *Scutellaria baicalensis charcoal (Chao huangqin)*,^24^ *Sophora flavescens (Ku shen)*,^25^ and *Indigo Naturalis (Qing dai)*.^26^ The differential outcomes suggest that the therapeutic effect of BHE is not linear but is highly dependent on the distinct pathophysiology and intervention timing of the inflammatory context, indicating a complex mechanism of action that warrants further investigation.

UC is characterized by dysregulated intestinal inflammatory responses, where key inflammatory mediators play critical roles.^27^ Among these, TNF-α stands out as both a well-established therapeutic target and a crucial biomarker of UC progression. This understanding has directly led to the clinical development and widespread utilization of various monoclonal antibody therapies.^28^^,^^29^ Conversely, TGF-β, an immunosuppressive cytokine, exhibits significantly impaired activity in UC, making strategies to upregulate its expression a promising research avenue for therapeutic intervention.^30^^,^^31^ Similarly, IL-10, another common anti-inflammatory cytokine, holds potential therapeutic value in UC management.^32^^,^^33^ Notably, a case report detailed a patient whose condition failed to improve despite treatment with intravenous steroids and anti-TNF-α therapy. Ultimately, this patient underwent Fecal Microbiota Transplantation (FMT), which resulted in a significant amelioration of UC symptoms. Furthermore, a marked increase in the patient’s gut microbial diversity was observed post-FMT.^34^ This outcome strongly suggests an intricate relationship between the gut microbiota and intestinal inflammatory cytokines, highlighting the microbiota as a potentially pivotal factor in the modulation and suppression of intestinal inflammation.

Beyond macroscopic and inflammatory improvements, our investigation revealed significant differences in gut microbiota composition between AUC, CUC mice, and the normal control (K) group. While alpha diversity indices were similar (Figure 4A), beta diversity showed distinct variations (Figures 4B and 4C), indicating differences in dominant species distribution. Specifically, AUC_M was characterized by increased *norank_f_Muribaculaceae* and *Bacteroides* and decreased *Allobaculum* and *Dubosiella*. CUC_M exhibited a significant increase in *norank_o__Clostridia_UCG-014* alongside a notable decrease in *Allobaculum* and *Dubosiella* (Figures 4D and 4E). LEfSe analysis and Kruskal-Wallis H test further confirmed the significant enrichment of *Bacteroides* and *Alloprevotella* in AUC_M group (Figures 4F and 4G). These distinct microbial profiles in AUC and CUC may reflect different pathological mechanisms and adaptive adjustments of microbial communities during disease progression.

BHE intervention effectively modulated these microbial dysbiosis patterns. In the AUC group, BHE mediated changes in specific bacterial genera without significantly affecting overall alpha diversity (Figures 5A–5C). Notably, *Lactobacillus* (AUC_D), *Turicibacter* (AUC_Z), and *Prevotellaceae_UCG-001* (AUC_G, AUC_Y) were significantly increased (Figures 5D–5G). PICRUSt2 functional prediction suggested that BHE might intervene in AUC by modulating these specific genera (Figure 5H). Similarly, in the CUC group, BHE influenced specific genera without altering overall alpha diversity (Figures 6A–6C). Significant elevations were observed in *Rikenellaceae_RC9_gut_group* (CUC_G), *norank_f__Muribaculaceae*, *Akkermansia* (CUC_Y), *norank_o__Clostridia_UCG-014* (CUC_Z), and *norank_f__Muribaculaceae* (CUC_D) (Figures 6D–6G). PICRUSt2 analysis indicated alterations in the predicted function “N: Cell motility” following BHE intervention (Figure 6H), suggesting BHE’s potential to intervene in CUC by modulating these identified genera.

Notably, gut microbiota dysbiosis in acute UC may be related to infection triggers or overactive host immune responses, whereas microbial changes in chronic UC more often reflect an imbalance in niche competition caused by long-term inflammation.^35^ Studies have shown that in patients with UC, beneficial bacteria such as *Lactobacillus* significantly decrease,^36^ while opportunistic pathogens such as *Enterobacteriaceae* and *Escherichia* markedly increase, with this imbalance being particularly pronounced in the acute phase.^37^^,^^38^ This phenomenon can be attributed to the excessive activation of inflammatory responses during the acute phase, leading to drastic changes in the intestinal microecological environment, thereby inhibiting the growth of commensal bacteria and promoting the proliferation of pathogenic bacteria. BHE intervention demonstrated a more significant ability to modulate microbiota in the chronic stage, suggesting its potential suitability for long-term UC management. Furthermore, this study found a close association between *Bacteroides* and mucus barrier repair, consistent with recent reports on *Bacteroides* improving intestinal mucosal integrity,^39^ which further supports the potential of BHE to achieve precise regulation by targeting specific microbial communities.^40^ Zhang et al. have found that the abundance of *norank_o__Clostridia_UCG-014* is positively correlated with both colitis and pro-inflammatory factors, which is also consistent with our AUC and CUC model results.^41^ Finally, PICRUSt2 functional prediction analysis revealed alterations in several predicted microbial functions following BHE intervention. Notably, changes were observed in “M: Cell wall/membrane/envelope biogenesis” and “K: Transcription” in the AUC group (Figure 5H), as well as in “N: Cell motility” in the CUC group (Figure 6H).

Our study further validated BHE’s regulatory effect on colonic barrier function in both AUC and CUC. BHE intervention suppressed the expression of TNF-α, which reduced inflammation-induced damage to the intestinal mucosa, an effect particularly evident in AUC mice. Concurrently, BHE intervention upregulated the expression levels of mechanical barrier-related protein Occludin^42^ and the key mucus barrier component MUC2^43^ in the colonic tissues of both AUC and CUC mice (Figures 7 and 8), with this regulatory effect being particularly significant in the chronic phase, which accelerates the intestinal mucosal repair process and maintaining barrier stability. Additionally, BHE’s regulatory effect on gut microbiota may also indirectly contribute to the recovery of colonic barrier function, for example, by increasing the number of beneficial bacteria to reduce intestinal mucosal permeability and subsequently decrease pathogenic invasion. These results collectively indicate that BHE intervention not only improves the symptoms in AUC and CUC mice but also repairs colonic barrier function at the molecular level, offering new insights for the long-term management of UC.

The findings of this study align with previous similar research while introducing unique innovations. Firstly, concerning alterations in gut microbiota, this study observed a reduction in beneficial bacteria (probiotics) and an increase in harmful bacteria in the intestines of AUC and CUC subjects, which is largely consistent with previous studies. However, this study further elucidated the dynamic changes in microbial composition and abundance across various disease stages using 16S rRNA gene sequencing technology, thereby providing more refined data to enhance our understanding of UC’s pathological mechanisms. Nevertheless, the influence of gender on these findings warrants further investigation. Secondly, concerning the efficacy of BHE intervention, previous studies have primarily focused on single formulas or components. This study, however, systematically evaluated the comprehensive effects of a Traditional Chinese Medicine (TCM) compound formula on gut microbiota and the colonic barrier. Importantly, by integrating 16S rRNA gene sequencing with immunofluorescence techniques, we simultaneously investigated the interplay between microbial changes and colonic barrier function. This methodological innovation offers a novel reference framework for future research.

In conclusion, this study uncovered distinct gut microbiota characteristics in the AUC and CUC models, mirroring the dynamic changes throughout disease progression. BHE exhibited unique benefits in treating UC by modulating the gut microbiota and restoring intestinal barrier function, providing new insights for refining TCM formula design and clinical application.

### Limitations of the study

To overcome these challenges and advance the investigation, upcoming studies should focus on large-scale human clinical trials involving a larger group of participants to comprehensively assess the effectiveness and safety of BHE. Furthermore, employing more advanced sequencing platforms such as metagenomics, metatranscriptomics, or longer-read technologies could provide more accurate microbial classification information and functional prediction results. Concurrently, integrating approaches such as metabonomics and network pharmacology would help elucidate the precise targets and interaction mechanisms of key components within the herbal formula.

## Resource availability

### Lead contact

Further information and requests for resources and reagents should be directed to and will be fulfilled by the lead contact, Wenjing Pei (peisuper@163.com).

### Materials availability

This study did not generate new unique reagents.

### Data and code availability

The 16S rRNA sequencing datasets for the AUC and CUC models generated during this study have been deposited into CNSA with accession number CNP0007913. All other data supporting the findings of this study are available from the lead contact upon reasonable request.

## Acknowledgments

We sincerely thank all researchers for their invaluable assistance and the participants for their valuable contributions to this study. The findings from this research are intended for dissemination through academic publications. We extend our grateful thanks to Majorbio Cloud platform (https://cloud.majorbio.com) for providing the sequencing and bioinformatics analysis services. This study received financial support from the “Jiebang Guashuai” Project of 10.13039/501100004846Beijing University of Chinese Medicine (Project No. 2022-JYB-JBZR-043, 2022-JYB-JBZR-008), Key Discipline of High-level Traditional Chinese Medicine of the National Administration of Traditional Chinese Medicine: Clinical Integration of Traditional Chinese and Western Medicine (Gastroenterology) (No. zyyzdxk-2023271), National High Level Chinese Medicine Hospital Clinical Research Funding (2025XZYJ04) and 2024 China Association of Chinese Medicine Youth Qiu-Shi Project (2024-QNQS-17).

## Author contributions

Wenjing Pei and Xiaowei Chen provided overall leadership and supervision throughout the project. Juncong Hu and Liming Zhang drafted the article. Data analysis was conducted by Zhibin Wang, Lei Shi, Yang Zhang, and Tangyou Mao. Juncong Hu, Liming Zhang, Yang Zhang, and Xiaowei Chen analyzed the primary sample and provided bioinformatics support. All authors give final approval to the submitted version.

## Declaration of interests

The authors declare they have no conflicts of interest.

## Declaration of generative AI and AI-assisted technologies in the writing process

During the preparation of this work, the authors did not utilize any AI or AI-assisted technologies.

## STAR★Methods

### Key resources table

**Table:** 

REAGENT or RESOURCE	SOURCE	IDENTIFIER
**Antibodies**		

anti-occludin	servicebio	Cat# GB111401; RRID:AB_2938979
anti-MUC2	servicebio	Cat# GB11344; RRID:AB_3082982

**Critical commercial assays**		

Mouse TNF-α Uncoated ELISA Kit	Thermo Fisher Scientific	Cat# 88-7324-88
Mouse/Human TGF-β ELISA Kit	Thermo Fisher Scientific	Cat# 88-8350-86
Mouse IL-10 Uncoated ELISA Kit	Thermo Fisher Scientific	Cat# 88-7105-88
FastPure Fecal DNA Extraction Kit	Shanghai MJYH	N.A.
SMRTbell Preparation Kit 3.0	PacBio Biosciences	N.A.
Fecal Occult Blood Reagent	BASO	B240701

**Experimental models: Organisms/strains**		

C57BL/6J mice	SPF (Beijing) Biotechnology Co., Ltd.	N.A.

**Software and algorithms**		

SMRT Link v11.0	Pacibo	https://www.pacb.com/smrt-link/
Silva v138	N.A.	http://www.arb-silva.de/
PICRUSt2	The Huttenhower lab	https://huttenhower.sph.harvard.edu/picrust/
LEfSe	The Huttenhower lab	https://huttenhower.sph.harvard.edu/lefse/
Mothur v1.30.1	N.A.	https://mothur.org/
Vegan v2.5-3	N.A.	https://vegandevs.github.io/vegan/
Prism 9.5.0 for macOS	GraphPad	https://www.graphpad.com/features

**Other**		

DSS	MP	Cat# 160110
Paeoniflorin reference standard	National Institutes for Food and Drug Control (NIFDC)	Batch# 110736-202246
BHE samples	Anhui Jiasen Pharmaceutical Technology Co., Ltd.	Batch# 230401, 230402, 230403
Majorbio Cloud platform	Shanghai Majorbio Biomedicine Technology Co., Ltd.	https://cloud.majorbio.com

**Deposited data**		

AUC Mice 16s rRNA-seq	This paper	Deposited into CNSA with accession number CNP0007913
CUC Mice 16s rRNA-seq	This paper	Deposited into CNSA with accession number CNP0007913

### Experimental model and study participant details

#### Mice

The animals used in this study were all of the C57BL/6 background. Four to six week old male C57BL/6J mice, weighing approximately 20 ± 2 grams, were bred in specific pathogen-free (SPF) conditions and sourced from SPF (Beijing) Biotechnology Co., Ltd. The animals were housed in a pathogen-free environment at Beijing University of Chinese Medicine, where environmental parameters were strictly controlled. The temperature was maintained at 22 ± 2°C, humidity levels were kept between 50% and 60%, and a 12-hour light-dark cycle was established.

### Method details

#### HPLC analysis of BHE active ingredients

Paeoniflorin, identified as a major active ingredient in BHE, was quantified using a High-Performance Liquid Chromatography (HPLC) method. Paeoniflorin reference standard (Batch No. 110736-202246, purity 96.7%) was obtained from the National Institutes for Food and Drug Control (NIFDC), China. BHE samples (Batch Nos. 230401, 230402, 230403, 50 mL/bottle) were provided by Anhui Jiasen Pharmaceutical Technology Co., Ltd., China. HPLC-grade acetonitrile and methanol, along with analytical-grade phosphoric acid, were utilized for the analysis. Chromatographic separation was performed using a HPLC instrument equipped with an ultraviolet/diode array detector and a C18 column. The mobile phase consists of acetonitrile and 0.1% phosphoric acid solution in a fixed ratio (15:85, volume ratio), and is delivered isocratically at a flow rate of 1.0 ml per minute. Detection was performed at a wavelength of 230 nm, the column temperature was maintained at 30 degrees Celsius, and the injection volume was used for analysis at 10 μL.

Paeoniflorin standard solutions were prepared by accurately weighing the reference standard and diluting it with methanol to create a series of concentrations for a calibration curve. BHE samples were prepared by accurately measuring a specific volume, dissolving it in 80% methanol, and filtering prior to injection. System suitability was confirmed by assessing parameters such as theoretical plate number, retention time, peak area RSD, and tailing factor, ensuring the method’s robustness. Paeoniflorin was identified by retention time comparison with the standard. Quantification was measured by external standard technology, using a linear calibration curve drawn over an appropriate concentration range with R^2^ values exceeding 0.999. This calibration curve was then used to determine the content in BHE samples.

#### Animal experimental design

Male C57BL/6J mice, aged four to six weeks and weighing approximately 20 ± 2 grams, had unrestricted access to an SPF-grade standard diet and deionized water. Following a one-week acclimation period, during which each mice was housed individually, they were utilized for experimental procedures.

In the mice models of acute ulcerative colitis (AUC), all experimental groups except the control group induced colitis by allowing mice to drink water containing 2.5% sodium dextran sulfate (DSS) *ad libitum* for seven days with the BHE intervention performed concurrently. AUC_M model group (which did not receive any intervention), Bawei Huangqin Enema high-dose (AUC_G, 1.8096 g/mL), Bawei Huangqin Enema medium-dose (AUC_Z, 0.9048 g/mL), Bawei Huangqin Enema low-dose (AUC_D, 0.4524 g/mL), and a positive control group receiving Mesalazine enema (AUC_Y, Salofalk) at a dose of 455 mg/(kg·d). Treatments were administered four times per week at a consistent daily time. To collect colon samples for subsequent HE, ELISA, and immunohistochemistry assays, all mice were anesthetized with isoflurane at the end of the experiment.

For the chronic UC (CUC) mice models, induction involved three cycles. Each cycle consisted of 6 days of *ad libitum* 2% DSS solution followed by 6 days of *ad libitum* deionized water. Upon successful induction of the CUC model, those mice that had received induction were randomly divided into five different experimental groups, CUC_M model group (which did not receive any intervention), Bawei Huangqin Enema high-dose (CUC_G, 1.8096 g/mL), Bawei Huangqin Enema medium-dose (CUC_Z, 0.9048 g/mL), Bawei Huangqin Enema low-dose (CUC_D, 0.4524 g/mL), and a positive control group receiving Mesalazine enema (CUC_Y, Salofalk) (455 mg/(kg·d)). Including the healthy control group, this also resulted in a total of six groups. Enema administration began on the first day after successful model induction. Treatments were administered four times per week for two consecutive weeks at a consistent daily time. At the end of the study, all mice were anesthetized with isoflurane to facilitate the collection of colon tissue samples, which were subsequently used for hematoxylin-eosin staining, ELISA, and immunohistochemical analysis.

The Institutional Animal Care and Use Committee of Beijing University of Chinese Medicine approved all animal experiments and procedures, which were conducted under protocol BUCM-2024061503-2345 and BUCM-2024080102-3130.

#### DAI score assessment

To quantitatively evaluate disease progression, we monitored the mice daily for key clinical indicators, including body weight changes, stool consistency, and the presence of blood in the stool. Fecal blood was specifically identified using a Fecal Occult Blood Reagent (BASO, B240701). To assess clinical progression during the BHE intervention, a daily Disease Activity Index (DAI) was computed for 6 mice per group using a composite scoring system. This system assigned scores from 0 to 4, where a score of 0 represented a healthy state with no weight loss, well-formed stool, and no detectable blood. Progressive scores were assigned for increasing severity: a score of 1 for 1-5% weight loss; a score of 2 for 5-10% weight loss, the presence of semi-formed stool, or a positive occult blood test; a score of 3 for 10-15% weight loss; and a maximum score of 4 for severe indicators such as weight loss greater than 15%, liquid stool, or the presence of visible blood.^44^^,^^45^

#### Histological injury scores assessment

For the histopathological assessment of colonic damage, tissue samples were fixed in 10% neutral buffered formaldehyde, subjected to routine processing, and embedded in paraffin. Subsequently, 5-μm-thick sections were cut using a rotary microtome and stained with HE. The stained sections were then examined via light microscopy for a semi-quantitative evaluation of key pathological indicators, including ulceration, crypt damage, congestion, edema, and inflammatory infiltration. Following established methodologies,^46^ a composite histological sore (maximum of 13) was determined for each sample based on the assessment of at least four distinct microscopic fields. Finally, representative photomicrographs were taken at various magnifications to document the histological changes.

#### DNA extraction and PCR amplification

Fecal samples were collected from each group of mice 7 days after enema treatment (AUC model) and 14 days after enema treatment (CUC model), and then 16s rRNA gene sequencing was performed to assess microbial diversity. Microbial genomic DNA was then isolated from these fecal specimens using the FastPure Fecal DNA Extraction Kit (Shanghai MJYH, China) following the manufacturer’s detailed instructions. DNA quality was verified on a 1% agarose gel, and its quantity and purity were determined using a NanoDrop2000 spectrophotometer (Thermo Fisher Scientific, Inc., USA). To analyze bacterial communities, the 16s rRNA gene was amplified using universal primers 27F (5′-AGRGTTYGATYMTGGCTCAG-3′) and 1492R (5′-RGYTACCTTGTTACGACTT-3′). These primers were modified with PacBio barcode sequences to identify individual samples. Each PCR reaction with a total volume of 20 microliters contained 4 microliters of 5X FastPfu buffer, 2 microliters of 2.5 mM dNTPs, 0.8 microliters of each primer (5 μM), 0.4 microliters of FastPfu DNA polymerase, 10 nanograms of template DNA, and made up to the final volume with nuclease-free water. The amplification process on a T100 thermal cycler (Burr, USA) consisted of initial denaturation at 95°C for 3 minutes, 27 cycles at 95°C for 30 seconds each, 60°C for 30 seconds, 72°C for 45 seconds, and finally extension at 72°C for 10 minutes and storage at 4°C. After electrophoresis, the PCR products were purified using AMPure® PB magnetic beads (Pacific Biosciences, USA) and quantified using Qubit 4.0 equipment (Thermo Fisher Technologies, USA).

#### DNA library preparation and sequencing process

The purified DNA fragments are mixed in an equal molar ratio. Library preparation was then carried out using the SMRTbell Preparation Kit 3.0 (PacBio Biosciences, California, USA), strictly following the precise guidelines provided by PacBio. These SMRTbell libraries were then sent to Meiji Biomedical Technology Co., Ltd. in Shanghai for sequencing, using the PacBio Sequel IIe system from PacBio Biosciences in California, USA. During the sequencing process, sub-reads are generated by circular consensus sequence sequencing (CCS) using SMRT Link v11.0 software, resulting in high-precision (HiFi) reads.

#### Data processing and statistical analysis

Initially, HiFi readings were assigned barcodes and filtered based on length. Bacterial 16s rRNA sequences that are less than 1000 base pairs or more than 1800 base pairs will be excluded, while fungal ITS sequences that are not in the range of 300 - 900 base pairs will also be discarded. were discarded. OTU representative sequences were classified taxonomically using RDP Classifier version 2.218 with a confidence threshold of 0.7, using 16s rRNA reference databases such as Silva v138.^47^ Alpha diversity (Shannon, Chao indices) was then calculated to assess community richness and diversity. Principal coordinate analysis (PCoA) and non-metric multidimensional scale analysis (NMDS) were used to delineate differences in sample or group structure. In order to statistically assess differences between groups, ANOSIM and Adonis tests were performed.

Drawing from the taxonomic annotation, stacked bar charts and heatmaps were generated. Visualizations present the composition of microbial communities at the genus level between different groups, focusing on changes in the composition and relative abundance of key species. Linear discriminant analysis of effect size (LEfSe)^48^ was also performed to identify bacterial groups with statistically significant differences in abundance, covering taxonomic levels from phylum to genus. The analyzed community abundance data were processed using reliable statistical techniques, including Kruskar-Wallis rank-sum test, one-way analysis of variance, and Wilcoxon rank-sum test. The purpose of these tests was to perform hypothesis testing on species abundance variations across microbial groups, ascertain the significance of observed differences, and identify species exhibiting significant differential abundance between groups.

Finally, metagenomic function prediction was carried out using PICRUSt2,^49^ leveraging the OTU representative sequences. PICRUSt2 incorporates several tools for this purpose: HMMER was used to align OTU representative sequences with reference sequences, while EPA-NG and Gappa were used to map these OTU sequences into the reference phylogenetic framework. Castor normalized the copy number of the 16s gene and applied MinPath to infer gene family characteristics and associate them with corresponding metabolic pathways. The entire analysis process is carried out in strict accordance with the standardized procedures specified in PICRUst2.

#### Elisa assay and immunofluorescence assay

Colon samples from each mice group were stored at -80°C for subsequent analysis. ELISA for TNF-α, TGF-β, and IL-10 was performed using specific kits from Thermo Fisher Scientific, USA: the Mouse TNF-α Uncoated ELISA Kit (# 88-7324-88), Mouse/Human TGF-β ELISA Kit (# 88-8350-86), and Mouse IL-10 Uncoated ELISA Kit (# 88-7105-88). Following the manufacturer’s instructions, samples and reagents were sequentially added, followed by incubation, washing, and colorimetric detection steps. Optical density values for TNF-α, TGF-β, and IL-10 were determined, and their respective concentrations were calculated using an enzyme marker. For the analysis of Occludin and MUC2, an immunofluorescence assay was employed. Tissue specimens were preserved with 4% paraformaldehyde, then embedded in paraffin and cut into 4 micron sections, used citrate buffer for antigen retrieval, and then the samples were permeabilized and blocked. After incubation with the primary and secondary antibodies, the sections were finally imaged. Primary antibodies used included anti-occludin (Mouse; servicebio; cat. no.GB111401; 1:200) and anti-MUC2 (Mouse; servicebio; cat. no.GB11344; 1:500). Whole-slide fluorescent imaging was performed using a Pannoramic MIDI (3DHISTECH).

### Quantification and statistical analysis

#### Statistical analysis

Bioinformatic analysis of the gut microbiota was conducted leveraging the Majorbio Cloud platform (https://cloud.majorbio.com). Utilizing the OTU data, rarefaction curves and various alpha diversity metrics, such as observed OTUs, Chao richness, and the Shannon index, were computed using Mothur v1.30.1.^50^ Community similarities across different samples were assessed through principal coordinate analysis (PCoA), which was based on Bray-Curtis dissimilarity and implemented with the Vegan v2.5-3 package. To pinpoint bacterial taxa (from phylum to genus level) exhibiting significant differential abundance across the distinct groups, Linear Discriminant Analysis (LDA) Effect Size (LEfSe) (http://huttenhower.sph.harvard.edu/LEfSe) was employed.^48^ Notably, specific thresholds were applied: an LDA score > 2 with *p* < 0.05 for the AUC group, and an LDA score > 3.5 with *p* < 0.05 for the CUC group. Data from animal experiments are presented as the mean ± standard deviation, derived from a minimum of three independent experimental replicates. All statistical analyses were conducted using Prism 9 for macOS software (Version 9.5.0). Group comparisons were performed using the Kruskal-Wallis H test, with statistical significance set at *p* < 0.05.
