# Supplementary material for: Modulation of distinct gut microbiota signatures in acute and chronic DSS-colitis by Bawei Huangqin enema in mice
Source: iScience. 2025 Sep 12;28(10):113568. doi: 10.1016/j.isci.2025.113568 (PMC12539248; doi:10.1016/j.isci.2025.113568)
Supplement: Document S1. Figures S1–S3 [file mmc1.pdf]

iScience, Volume *28*

Supplemental information

Modulation of distinct gut microbiota signatures in acute and chronic DSS-colitis by Bawei Huangqin enema in mice

Juncong Hu, Liming Zhang, Zhibin Wang, Lei Shi, Yang Zhang, Tangyou Mao, Xiaowei Chen, and Wenjing Pei

Supplementary Materials Catalogs

Figure S1 The boxplot of chao index between AUC and CUC.
Figure S2 The boxplot of chao index in AUC related intervention.
Figure S3 The boxplot of chao index in CUC related intervention.

Figure S1 The boxplot of chao index between AUC and CUC.

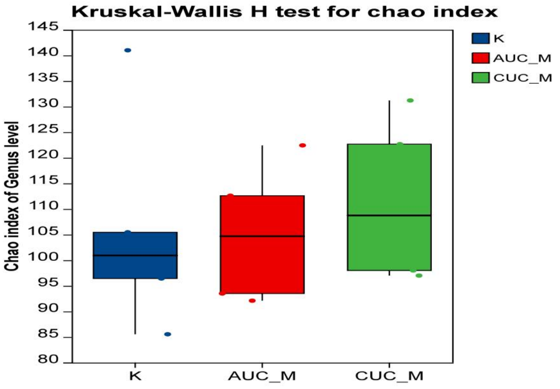

Figure S1. The boxplot of chao index between AUC and CUC. K, control group, AUC_M, acute colitis model group, CUC_M, chronic colitis model group.

Figure S2 The boxplot of chao index in AUC related intervention.

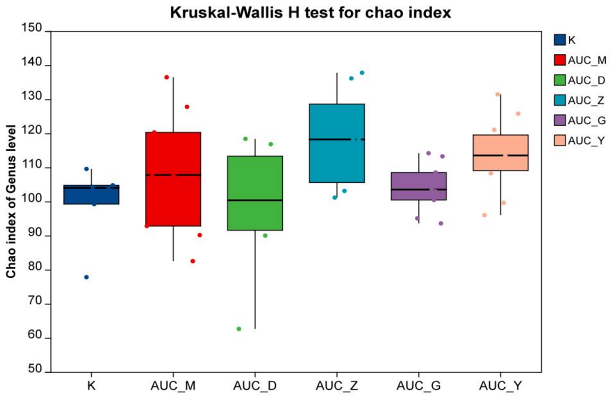

Figure S2. The boxplot of chao index in AUC related intervention. K, control group, AUC_M, acute colitis model group, AUC_Y, mesalazine enema group, AUC_D, low-dose BHE group, AUC_Z, medium-dose BHE group, AUC_G, high-dose BHE group.

Figure S3 The boxplot of chao index in CUC related intervention.

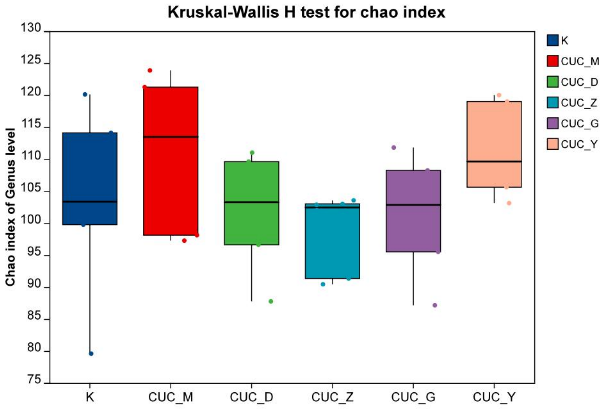

Figure S3. The boxplot of chao index in CUC related intervention. K, control group, CUC_M, chronic colitis model group, CUC_Y, mesalazine enema group, CUC_D, low-dose BHE group, CUC_Z, medium-dose BHE group, CUC_G, high-dose BHE group.
